# Effectiveness of a treat-to-target strategy in patients with moderate to severely active rheumatoid arthritis treated with abatacept

**DOI:** 10.1186/s13075-023-03151-2

**Published:** 2023-09-28

**Authors:** Louis Bessette, Boulos Haraoui, Emmanouil Rampakakis, Joanna Dembowy, Marc-Olivier Trépanier, Janet Pope

**Affiliations:** 1 Department of Medicine, Laval University, Quebec, QC Canada; 2 Centre Hospitalier de L’Université de Montréal, Montreal, Québec Canada; 3 Department of Pediatrics, McGill University, Montreal, Canada; 4 JSS Medical Research, Montreal, Canada; 5 Brisol Myers Squibb, Montreal, Québec Canada; 6 Division of Rheumatology, Department of Medicine, Western University, 268 Grosvenor Street, London, ON N6A 4V2 Canada

**Keywords:** Abatacept, Real-world, Rheumatoid arthritis, Routine care, Treat-to-target

## Abstract

**Background:**

To compare a treat-to-target (T2T) approach and routine care (RC) in adults with active to severely active rheumatoid arthritis (RA) initiating subcutaneous abatacept.

**Methods:**

A 12-month cluster-randomized trial in active RA patients treated with abatacept was conducted. Physicians were randomized to RC or T2T with a primary endpoint of achieving sustained Clinical Disease Activity Index (CDAI) low disease activity (LDA) at two consecutive assessments approximately 3 months apart. Additional outcomes included Simple Disease Activity Index (SDAI), Disease Activity Score 28-CRP (DAS28-CRP), Routine Assessment of Patient Index Data 3 (RAPID3), and the Health Assessment Questionnaire-Disability Index (HAQ-DI). Time to achieve therapeutic endpoints was assessed with survival analysis.

**Results:**

Among the 284 enrolled patients, 130 were in the T2T group and 154 in RC. Primary endpoint was achieved by 36.9% and 40.3% of patients in T2T and RC groups, respectively. No significant between-group differences were observed in the odds of achieving secondary outcomes, except for a higher likelihood of CDAI LDA in the T2T group vs. RC (odds ratio [95% confidence interval]: 1.33 [1.03–1.71], *p* = 0.0263). Compared with RC, patients in the T2T group achieved SDAI remission significantly faster (Kaplan–Meier-estimated mean [standard error]: 14.0 [0.6] vs. 19.3 [0.8] months, *p* = 0.0428) with a trend toward faster achievement of CDAI LDA/remission, DAS28-CRP remission, and HAQ-DI minimum clinically important difference.

**Conclusions:**

Patients managed per T2T and those under RC experienced significant improvements in RA disease activity at 12 months of abatacept treatment. T2T was associated with higher odds of CDAI LDA and a shorter time to achieving therapeutic endpoints.

**Trial registration:**

Name of the registry: ClinicalTrials.gov.

Trial registrations: NCT03274141.

Date of registration: September 6, 2017.

**Supplementary Information:**

The online version contains supplementary material available at 10.1186/s13075-023-03151-2.

## Background

Rheumatoid arthritis (RA) is a common autoimmune disease characterized by a progressive inflammatory synovitis of the joints, leading to cartilage and bone damage as well as disability [1, 2]. According to the 2017 Global Burden of Disease, Injuries, and Risk Factors Study, the age-standardized global RA prevalence is 246.6 per 100,000 with an incidence of 14.9 per 100,000 [3].

RA is associated with severe functional disability, reduction in health-related quality of life (HRQoL) and an increase in premature mortality [4, 5]. Therapeutic options currently available for treatment of RA include non-steroidal anti-inflammatory drugs (NSAIDs), glucocorticoids, and disease-modifying anti-rheumatic drugs (DMARDs). Conventional DMARDs are the recommended first-line therapy for RA with methotrexate (MTX) monotherapy as the preferred option [6, 7]. In patients who fail first-line treatment, biologic DMARDs (bDMARDs), such as tumor necrosis factor inhibitors (TNFi), anti-IL-6 agents, T cell activation blockade or targeted synthetic DMARDs (tsDMARDs), including Janus kinase inhibitors (JAKi), are recommended [6, 7]. Abatacept, a bDMARD, is a fully human recombinant fusion protein consisting of cytotoxic T lymphocyte–associated protein 4 that binds to CD80/CD86 on antigen-presenting cells and activated T cells, thereby outcompeting CD28 present on T cells, a CD80/86 substrate and a co-stimulatory molecule required for maximal T cell activation. Disruption of CD28 interactions with CD80/86 effectively downregulates T cell activation and may result in anergy and apoptotic cell death [8]. The safety and efficacy of abatacept in reducing RA disease activity and improving patient reported outcomes (PROs) has been demonstrated in several clinical trials [9–11] and observational studies [12–15].

The treat-to-target (T2T) approach is a shared decision-making process whereby strict management of a particular treatment course is directly influenced by patient disease activity scores in relation to a treatment target such as remission [16, 17]. Evidence in support of the T2T approach as compared to early, aggressive treatment strategies includes better disease control and improved long term outcomes in RA patients [18–21]. Less is known about the T2T approach compared to routine care (RC) when an advanced therapy is initiated [22]. Here we report the results of the Abatacept Best Care (ABC) trial (NCT03274141), a prospective, observational study aimed at comparing the T2T approach vs. RC in real-life management of adult patients with active to severely active RA initiating subcutaneous (SC) abatacept as first- or second-line biologic agent.

## Methods

### Data source and study design

The ABC trial was a 12-month prospective, multicenter, post-marketing, cluster (at the physician level) randomized study, with the option to participate in a 12-month extension phase for patients who completed the initial 12-month study. Physicians were randomized in a 1:1 ratio to either receive formal education on the T2T approach (T2T group) prior to the study or not receive training and instead continue managing through their judgment and RC (RC group). Physicians in the T2T group were provided with a thorough review of the T2T approach by T2T experts, and were encouraged to follow suggested treatment optimization options, as well as the Canadian Rheumatology Association Recommendations for Pharmacological Management of Rheumatoid Arthritis with Traditional and Biologic DMARDs [23], which suggest the goal of treatment should be remission or, when not possible, minimal disease activity, and provide recommendations about how to reach this goal (i.e., frequency of disease activity monitoring and use of concomitant medications). As the enrolled patients were at different stages of their disease and there was no consensus on the specific outcome to be targeted, a treatment algorithm was not imposed in the T2T group, rather physicians were merely informed of targets to be reached. In addition to this training, physicians in the T2T group received emails and newsletters every 2 months as a reminder of the guidelines. In line with the observational nature of the study, there were no pre-determined protocol-defined requirements for follow-up schedules; physicians managed patients in accordance with the current standard of care and regional requirement. Follow-up visits were recommended and encouraged at least every 3 (± 1) months during the first year of follow-up, and every 6 (± 1) months during the second year. Assuming a sustained Clinical Disease Activity Index (CDAI) low disease activity (LDA) rate for the RC group of 40%, and an odds ratio (OR) of 2.0 in favor of T2T, approximately 145 patients per group were required to achieve a statistical power of 80% and a 5% significance level.

#### Patients

Adult patients (≥ 18 years of age) with moderate-to-severe RA, defined as CDAI > 10, who were to initiate treatment with SC abatacept obtained via usual care as per judgement of the treating physician, prior to and independently of consideration for study enrolment, were eligible for enrollment. Additional inclusion criteria were: ≥ 18 years of age; moderate to severely active RA, defined as CDAI > 10; provision of informed consent; and fulfillment of the reimbursement criteria for treatment with SC abatacept under provincial or private health insurance reimbursement coverage. Patients were excluded if they had received abatacept (SC or intravenous) prior to the enrolment visit; had failed more than 1 prior bDMARD therapy; and/or had a history of autoimmune disease or any joint inflammatory disease other than RA with the exception of concomitant secondary Sjogren’s syndrome.

#### Efficacy assessments

RA disease activity was assessed using the CDAI, the Simple Disease Activity Index (SDAI), and the Disease Activity Score 28-CRP (DAS28-CRP), all of which have previously been validated for use in RA. CDAI is based on the sum of tender and swollen joint counts (TJC and SJC) of 28 joints, patient global assessment (PtGA) of disease activity measured on a visual analogue scale (VAS, range 0–100 mm), and physician global assessment (MDGA, VAS 0–100 mm) [24]. CDAI scores range between 0 and 76 with a score ≤ 2.8 indicative of remission, LDA defined by scores > 2.8 and ≤ 10, moderate activity ranging between scores > 10 and ≤ 22, and scores > 22 indicative of high activity [25]. SDAI is calculated as the numerical sum of TJC and SJC, PtGA, MDGA, and C-reactive protein (CRP) level (mg/dL) with scores ranging from 0–86 where remission is defined as a score ≤ 3.3, LDA ranges between scores > 3.3 and ≤ 11, scores > 11 and ≤ 26 are indicative of moderate disease activity, and high disease activity is defined by scores > 40 [26, 27]. DAS28-CRP includes TJC and SJC, PtGA, and CRP level [28] with scores ranging between 0 and 9 where a score ≤ 2.6 is indicative of remission, LDA is defined by scores > 2.6 and ≤ 3.2, moderate activity ranging between scores > 3.2 and ≤ 5.1, and scores > 5.1 indicative of high activity [25].

PROs used in the study included measures of disease activity, physical function, pain, and fatigue. The Routine Assessment of Patient Index Data 3 (RAPID3) is a validated composite index of RA disease activity based on 3 PROs included in the American College of Rheumatology Core Set PRO components: Patient Physical Function/Disability, Patient Pain, and PtGA. Each domain is scored from 0 to 10, for a total score of 30 and disease activity categories include remission with a score ≤ 3, score > 3 and ≤ 6 for LDA, score > 6 and ≤ 12 indicative of moderate activity, and high activity defined by a score > 12 [29, 30]. The Health Assessment Questionnaire Disability Index (HAQ-DI) measures functional status with a total score between 0–3, with increasing scores indicative of worse functioning (0 indicates no functional impairment and 3 complete impairment) [31]. A change of 0.22–0.25 units in HAQ-DI has been defined as the minimum clinically important difference (MCID) [30, 31]. Levels of pain [32] and fatigue were measured using a VAS (0–100 mm).

#### Outcomes

The primary outcomes were the proportion of patients and time to achieving sustained LDA (CDAI ≤ 10), for 2 consecutive assessments conducted approximately 3 months apart over a 1-year observation period.

Secondary outcomes included (i) the proportion of patients and the time to achieve CDAI LDA (≤ 10) and remission (≤ 2.8), SDAI remission (≤ 3.3), DAS28-CRP LDA (< 3.2) and remission (< 2.6), RAPID 3 LDA (≤ 6) and remission (≤ 3), HAQ-DI MCID (Δ ≥ 0.22) at months 3, 6, 9, 12; (ii) the change in CDAI, SDAI, DAS28-CRP, HAQ-DI, RAPID3, TJC28, SJC28, pain, and fatigue from baseline to months 3, 6, 9, 12; (iii) number of changes in RA treatment; and (iv) the incidence of treatment-emergent adverse events (TEAEs), including serious (SAEs) and non-serious AEs.

#### Data analysis

Data analysis was conducted on the modified intent-to-treat (mITT) population including all patients receiving at least one dose of SC abatacept. Descriptive statistics including the mean and standard deviation (SD) for continuous variables and frequency distributions for categorical variables were produced for all patient demographic data and baseline disease characteristics. The proportion of patients achieving the primary endpoint (sustained CDAI LDA) was compared between groups with a cluster-correct (Rao-Scott) Chi-square test; non-responder imputation was used for missing data. Time to achieve the primary endpoint was assessed with a Cox proportional hazard model with shared-frailty to account for the cluster effect and adjusted for site (random effect), site size (number of patients enrolled at each site), and potential confounders identified from the baseline comparison of the treatment groups (prior bDMARD use, smoking status). Achievement of secondary endpoints was assessed with mixed effects logistic regression for repeated measures adjusted for the same covariates as for the primary endpoint. Time to achieve secondary endpoints was assessed with the Kaplan–Meier (K-M) estimator of the survival function and compared between groups with the log-rank test. Least squares mean (LSM) changes from baseline in CDAI, SDAI, DAS28-CRP, RAPID3, and HAQ-DI were estimated with mixed models for repeated measures (MMRM) adjusting for the same covariates as for the primary endpoint. In MMRM, missing data are predicted by the observed data via the model for the conditional mean. The mean (95% confidence interval [CI]) count of changes per patient in RA treatment (NSAIDS, non-biologic/biologic DMARDs, intra-articular steroids, SC abatacept dose), including treatment interval modifications, were compared between groups using a cluster-corrected Student’s t-test. Given the observational nature of the study, no adjustment for multiplicity took place. All statistical analyses were performed using SAS software (*version 9.2*, SAS Institute, Cary, NC).

## Results

A total of 44 physicians were randomized, 21 in the RC group and 23 in the T2T group. Among physicians in the T2T and RC groups, approximately 65% and 76% were male, with an average duration of rheumatology practice of approximately 21 and 25 years, respectively, and comparable geographic distribution. The average number of patients enrolled at each site was 6.5 (95% CI: 5–8), ranging from 1–26.

### Baseline demographics and disease characteristics

The mITT population comprised 284 patients of whom 130 and 154 were included in the T2T and RC groups, respectively. A total of 207 (72.9%) patients completed the 12-months follow-up period, 84 (64.6%) and 123 (79.9%) in the T2T and RC groups, respectively. Most patients in the overall population were female (74.3%) and Caucasian (91.5%), mean (SD) age was 60.1 (11.6) years, and approximately half were current (18.3%) or past (34.5%) smokers. Baseline demographics were generally comparable between the treatment groups, although the proportion of Caucasians was higher in the RC group (Table 1).

**Table 1:** Baseline patient and disease characteristics

	**Treat-to-Target** **(*N* = 130)**	**Routine Care** **(*N* = 154)**	**Total** **(*N* = 284)**
**Age (years), mean (SD)**	59.2 ± 11.8	60.8 ± 11.4	60.1 (11.6)
**Sex,** n (%)			
Female	97 (74.6)	114 (74.0)	211 (74.3)
Male,	32 (24.6)	40 (26.0)	72 (25.4)
Missing	1 (0.8)	0 (0.0)	1 (0.4)
**Race**, n (%)			
Caucasian	112 (86.2)	148 (96.1)	260 (91.5)
Black or African American	1 (0.8)	1 (0.6)	2 (0.7)
Asian	6 (4.6)	1 (0.6)	7 (2.5)
Hispanic	3 (2.3)	1 (0.6)	4 (1.4)
Native/Aboriginal	1 (0.8)	0 (0.0)	1 (0.4)
Other	6 (4.6)	3 (1.9)	9 (3.2)
Missing	1 (0.8)	0 (0.0)	1 (0.4)
**Smoking Status, n (%)**			
Current	27 (20.8)	25 (16.2)	52 (18.3)
Past	41 (31.5)	57 (37.0)	98 (34.5)
None	60 (46.2)	72 (46.8)	132 (46.5)
Missing	2 (1.5)	0 (0.0)	2 (0.7)
**Disease Characteristics**			
**RA duration (years)**			
Mean ± SD	7.9 ± 9.6	7.5 ± 8.6	7.7 ± 9.1
**RF status, n (%)**			
Negative	39 (30.0)	55 (35.7)	94 (33.1)
Positive	79 (60.8)	80 (51.9)	159 (56.0)
NA/Unknown/Not done	12 (9.2)	19 (12.3)	31 (10.9)
**Anti-CCP status, n (%)**			
Negative	32 (24.6)	41 (26.6)	73 (25.7)
Positive	48 (36.9)	42 (27.3)	90 (31.7)
NA/Unknown/Not done	50 (38.5)	71 (46.1)	121 (42.6)
**CRP status (mg/dl)**			
N (%)	109 (87.9)	128 (92.8)	237 (90.5)
Mean ± SD	1.7 ± 2.7	2.4 ± 4.3	2.1 ± 3.7
Median (IQR)	0.7 (0.2, 1.7)	0.9 (0.4, 2.5)	0.8 (0.3, 2.2)
**ESR (mm/hr)**			
N (%)	96 (100.0)	117 (100.0)	213 (100.0)
Mean ± SD	24.2 ± 20.1	22.1 ± 18.9	23.0 ± 19.5
Median (IQR)	19.0 (10.0, 31.5)	16.0 (9.0, 30.0)	17.0 (9.0, 30.0)
**MDGA (VAS 100 mm)**			
N (%)	123 (94.6)	152 (98.7)	275 (96.8)
Mean ± SD	64.3 ± 17.3	60.2 ± 16.9	62.0 ± 17.2
**TJC28**			
N (%)	126 (96.9)	150 (97.4)	276 (97.2)
Mean ± SD	9.5 ± 6.1	10.0 ± 6.1	9.8 ± 6.1
**SJC28**			
N (%)	126 (96.9)	153 (99.4)	279 (98.2)
Mean ± SD	7.7 ± 4.7	8.2 ± 4.5	8.0 ± 4.6
**Fatigue (VAS 100 mm)**			
N (%)	124 (95.4)	150 (97.4)	274 (96.5)
Mean ± SD	65.7 ± 21.9	60.8 ± 25.0	63.0 ± 23.7
**PtGA (VAS 100 mm)**			
N (%)	126 (96.9)	150 (97.4)	276 (97.2)
Mean ± SD	65.0 ± 21.4	61.9 ± 22.1	63.3 ± 21.8
**Pain (VAS 100 mm)**			
N (%)	126 (96.9)	150 (97.4)	276 (97.2)
Mean ± SD	66.3 ± 23.4	62.0 ± 24.3	64.0 ± 24.0
**HAQ-DI**			
N (%)	125 (96.2)	151 (98.1)	276 (97.2)
Mean ± SD	1.5 ± 0.6	1.5 ± 0.6	1.5 ± 0.6

*CCP* Cyclic citrullinated peptides, *CRP* C-reactive protein, *ESR* Erythrocyte sedimentation rate, *HAQ-DI* Health Assessment Questionnaire-Disability Index, *IQR* Interquartile range, *MDGA* Physician global assessment, *PtGA* Patient global assessment, *RA* Rheumatoid arthritis, *RC* Routine care, RF Rheumatoid factor, *SD* Standard deviation, *SJC* Swollen joint count, *T2T* Treat-to-target, *TJC* Tender joint count, *VAS* Visual analog scale

At baseline, patient disease parameters were similar between the treatment groups (Table 1). Overall, patients had a mean (SD) duration of RA of 7.7 (9.1) years, a mean (SD) CRP level of 2.1 (3.7) mg/dL, and a mean (SD) erythrocyte sedimentation rate (ESR) of 23.0 (19.5) mm/hr. Positive RF, and anti-CCP status was observed for 159 (56.0%) and 90 (31.7%) patients, respectively. The overall mean (SD) TJC28 (9.8 [6.1]), and SJC28 (8.0 [4.6]), as well as scores for fatigue (VAS: 63.0 [23.7]), MDGA (VAS: 62.0 [17.2]), pain (VAS: 64.0 [24.0]), PtGA (VAS: 63.3 [21.8]) and physical function (HAQ-DI: 1.5 [0.6]) were indicative of moderate to severe RA disease activity and disability.

Prior bDMARD use was reported in 54 (41.5%) and 57 (37.0%) patients in the T2T and RC groups, respectively (Table S1). Nearly all patients in both treatment groups reported concomitant non-bDMARD use at baseline, the most common being MTX (T2T: 70.0%, RC: 62.3%) and hydroxychloroquine (T2T: 36.2%, RC: 44.2%). NSAID use was comparable at baseline (T2T: 30.0%, RC: 27.9%); however, corticosteroids were more commonly used in the RC group (T2T: 18.5%, RC: 27.9%; Table S1).

### Clinical effectiveness

**Fig. 1:**
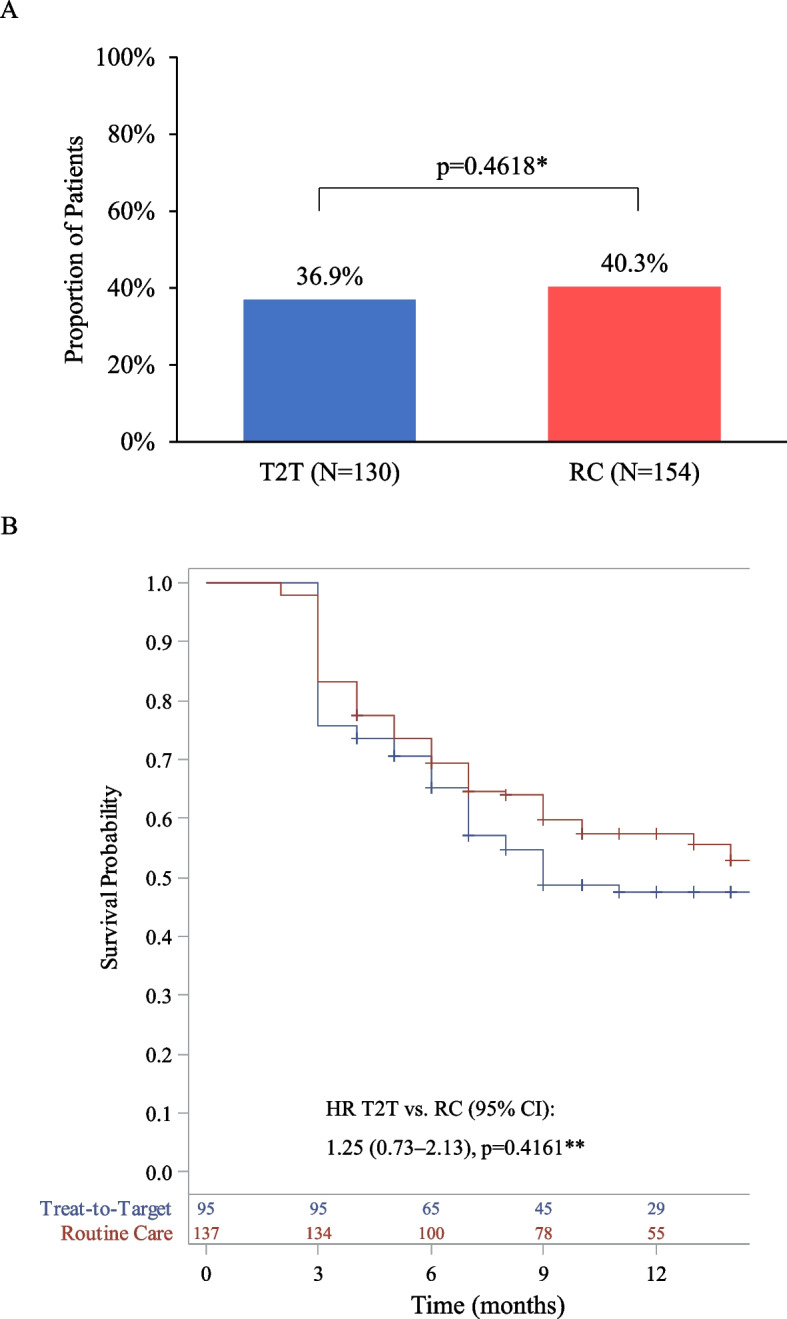
Achievement of sustained Clinical Disease Activity Index (CDAI) low disease activity (LDA). **A** Proportion of patients with sustained CDAI LDA; **B** Time to achievement of sustained CDAI LDA. *P-value was based on the clustered-corrected (Rao-Scott) Chi-square test. **P-value was based on Cox proportional hazard model adjusted for site (random effect), month, prior biologic disease-modifying antirheumatic drug exposure, site size, and smoking status. Crosses indicate censoring. CI: confidence interval; HR: hazard ratio; RC: routine care; T2T: treat-to-target

The primary outcome of sustained CDAI LDA was achieved by 36.9% vs. 40.3% of patients in the T2T vs. RC group, over the course of 12 months (*p* = 0.4618, Fig. 1a). Although not statistically different, the mean (standard error, SE) K-M estimated time to achieve sustained LDA was shorter for patients in the T2T group compared with those managed per RC (7.9 [0.4] vs. 11.8 [0.5] months, Fig. 1b).

**Fig. 2:**
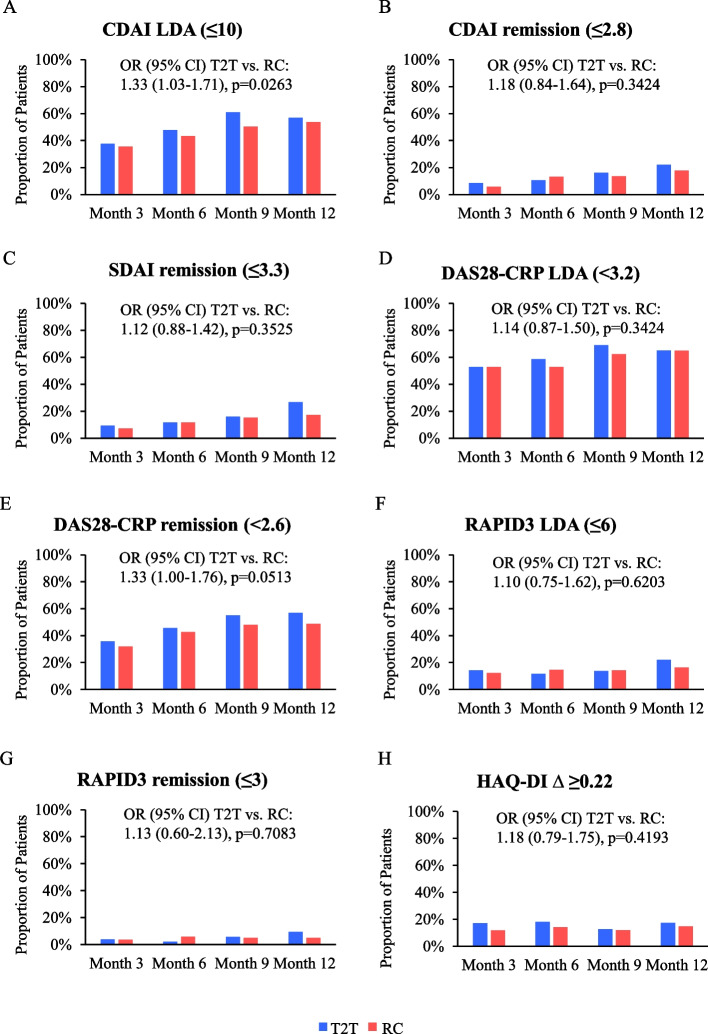
Achievement of secondary disease outcomes through 1 year. **A** CDAI LDA (≤ 10); **B** CDAI remission (≤ 2.8); **C** SDAI remission (≤ 3.3); **D** DAS28-CRP LDA (< 3.2); **E** DAS28-CRP remission (< 2.6); **F** RAPID3 LDA (≤ 6); **G** RAPID3 remission (≤ 3); **H** HAQ-DI Δ ≥ 0.22. Bars represent analyses of achievement of secondary outcomes as observed. At months 3, 6, 9, and 12, T2T sample size was 106, 93, 87, and 86, and RC sample size was 138, 136, 125, and 123, respectively. CDAI: Clinical Disease Activity Index; CI: confidence interval; DAS28-CRP: Disease Activity Score 28-C-reactive protein; HAQ-DI: Health Assessment Questionnaire-Disability Index; LDA: low disease activity; OR: odds ratio; RAPID3: Routine Assessment of Patient Index Data 3; RC: routine care; SDAI: Simple Disease Activity Index; T2T: treat-to-target

Analysis of secondary endpoints demonstrated that CDAI LDA at months 3, 6, 9 and 12 was achieved by 37.7%, 47.9%, 60.9%, and 57.0% of patients in the T2T group compared with 35.5%, 43.4%, 50.4%, and 53.7% of those managed per RC, while the rates of CDAI remission at months 3–12 ranged from 8.5–22.1% in the T2T group, and 5.8–17.9% in the RC group (Fig. 2a, b). Proportions of patients with SDAI remission at months 3–12 in the T2T and RC groups ranged from 9.4–26.7% and 7.2–17.1%, respectively (Fig. 2c). DAS28-CRP LDA at months 3 and 12 was achieved by 52.8% and 65.1% of patients in the T2T group, and 52.9% and 65.0% of those in the RC group, respectively, with DAS28-CRP remission at months 3, 6, 9, and 12 achieved by 35.8%, 45.7%, 55.2%, and 57.0% of patients in the T2T group compared with 31.9%, 42.6%, 48.0% and 48.8% of those in the RC group (Fig. 2d, e). In the T2T and RC regimens, proportions of patients with RAPID3 LDA ranged from 14.2–22.1% and 12.3–16.3% at months 3–12, respectively, and RAPID3 remission at months 3 and 12 was achieved by 3.8% and 9.3% of patients managed per T2T, and 3.6% and 4.9% of those managed per RC, respectively (Fig. 2f, g). Finally, 17.0%, 18.1%, 12.6%, and 17.4% of patients in the T2T group, and 11.6%, 14.0%, 12.0%, and 14.6% of those in the RC group achieved HAQ-DI MCID at months 3, 6, 9 and 12, respectively (Fig. 2h).

Logistic regression analysis of the odds of achieving secondary outcomes showed that compared with RC, patients in the T2T group were more likely to achieve CDAI LDA (OR [95% CI]: 1.33 [1.03–1.71], *p* = 0.0263; Fig. 2a). The likelihood of achieving all other secondary outcomes was statistically comparable between the regimens (Fig. 2).

**Fig. 3:**
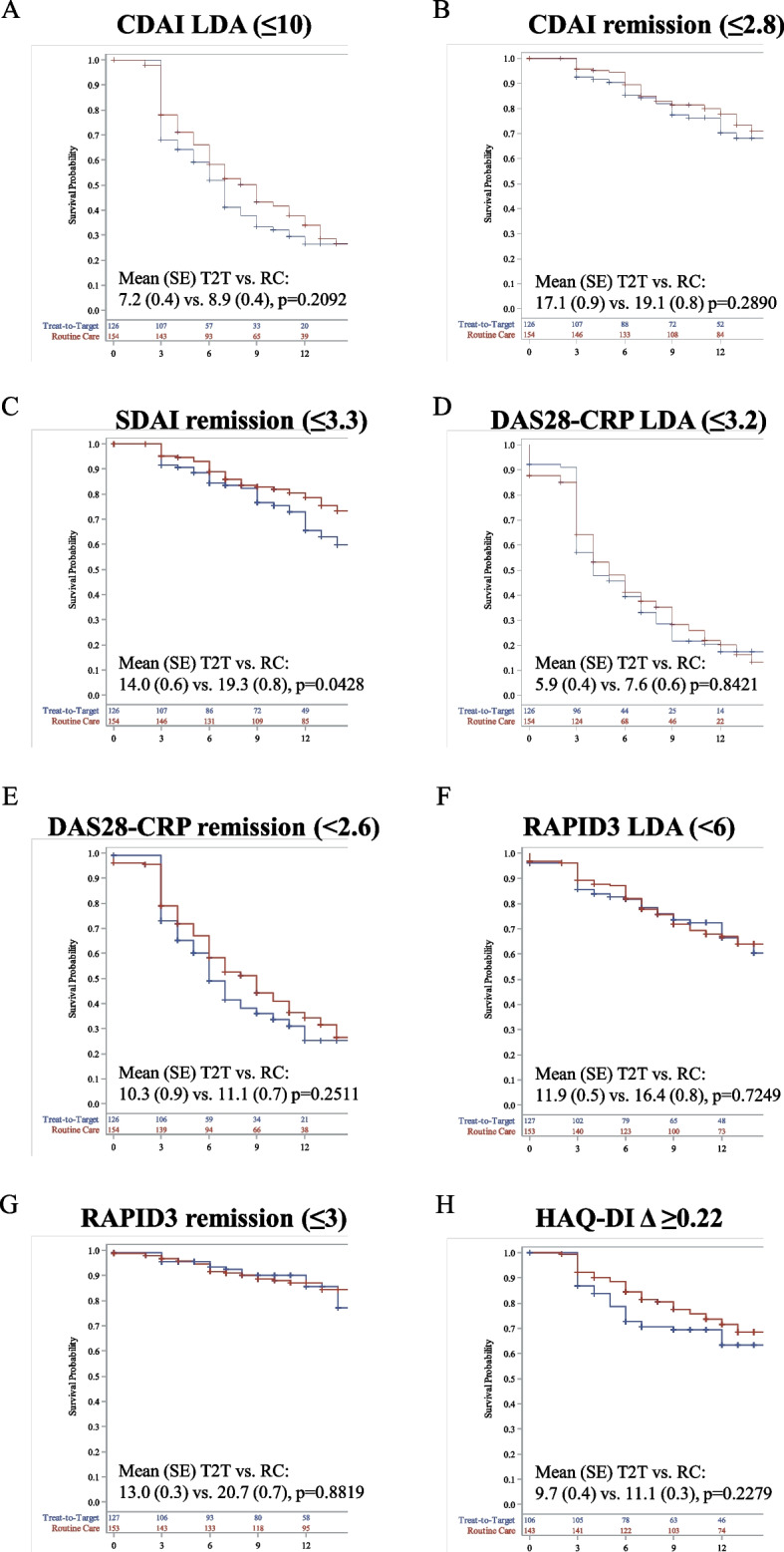
Time to achievement of secondary disease outcomes. **A** CDAI LDA (≤ 10); **B** CDAI remission (≤ 2.8); **C** SDAI remission (≤ 3.3); **D** DAS28-CRP LDA (< 3.2); **E** DAS28-CRP remission (< 2.6); **F** RAPID3 LDA (≤ 6); **G** RAPID3 remission (≤ 3); **H** HAQ-DI Δ ≥ 0.22. *P*-values are based on the log-rank test. Crosses indicate censoring. CDAI: Clinical Disease Activity Index; CI: confidence interval; DAS28-CRP: Disease Activity Score 28-C-reactive protein; HAQ-DI: Health Assessment Questionnaire-Disability Index; LDA: low disease activity; MCID: minimum clinically important difference; RAPID3: Routine Assessment of Patient Index Data 3; RC: routine care; SDAI: Simple Disease Activity Index; SE: standard error; T2T: treat-to-target

Survival analysis for the time to achieve secondary outcomes demonstrated that patients managed per T2T had a significantly shorter mean (SE) time to achieve SDAI remission than those treated through RC (K-M estimated: 14.0 [0.6] vs. 19.3 [0.8] months, *p* = 0.0428; Fig. 3c). The time to achieve all other secondary clinical outcomes and PROs was statistically comparable between the treatment groups, although a trend toward a faster achievement of CDAI LDA and remission, DAS28-CRP remission, and HAQ-DI MCID was observed in the T2T group (Fig. 3).

### Change in outcomes over time

**Fig. 4:**
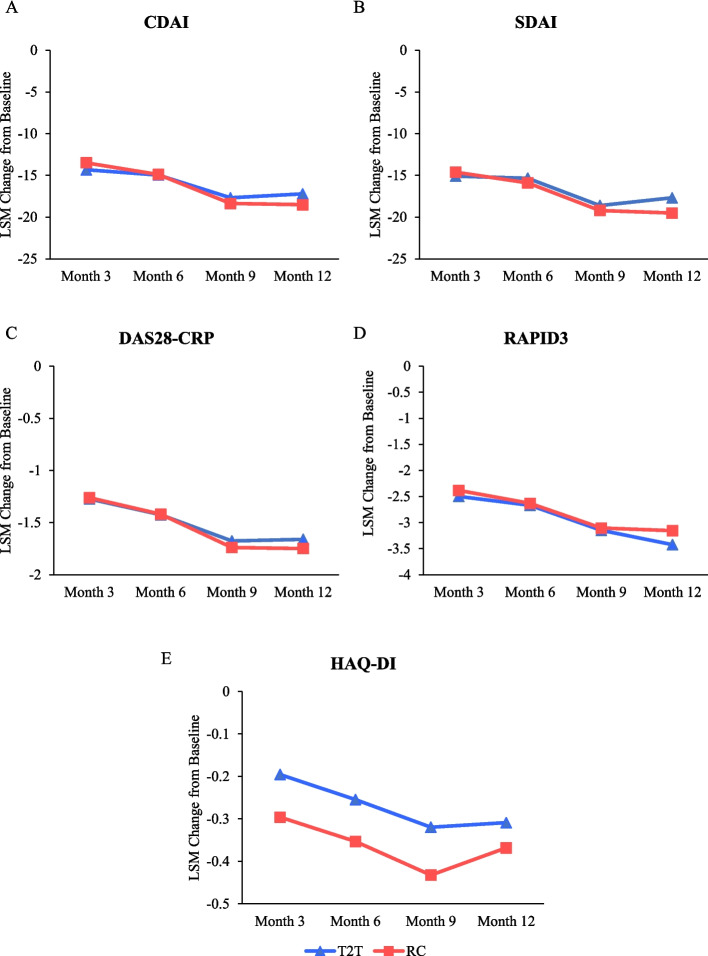
Least squares mean (LSM) change from baseline in disease outcomes through 1 year. **A** CDAI; **B** SDAI; **C** DAS28-CRP; **D** RAPID3; **E** HAQ-DI. All between-group comparisons were *p* > 0.05. All within-group differences from baseline over time were *p* < 0.01. CDAI: Clinical Disease Activity Index; DAS28-CRP, Disease Activity Score 28-C-reactive protein; HAQ-DI: Health Assessment Questionnaire-Disability Index; RAPID3: Routine Assessment of Patient Index Data 3; RC: routine care; SDAI: Simple Disease Activity Index; T2T: treat-to-target

LSM changes in CDAI, SDAI, DAS28-CRP, RAPID3 and HAQ-DI demonstrated statistically significant improvements from baseline within each treatment group (T2T and RC) as early as month 3 and were sustained through month 12 (*p* < 0.0001, Fig. 4). At month 12, LSM (SE) estimates corresponding to the change in CDAI, SDAI, DAS28-CRP, RAPID3, and HAQ-DI from baseline were -17.22 (1.58), -17.69 (1.61), -1.66 (0.17), -3.42 (0.64), and -0.31 (0.07) in the T2T group, respectively (*p* < 0.0001), and -18.52 (1.42), -19.51 (1.44), -1.75 (0.15), -3.15 (0.55), and -0.37 (0.06) in the RC group (Fig. 4).

In the overall population, the absolute mean change in disease measures and PROs from baseline to months 3–12 ranged from -5.3 to -6.5 for TJC28, -4.6 to -6.0 for SJC28, -14.5 to -21.2 mm for Pain, and -14.6 to -19.8 mm for Fatigue. Over time, no significant differences in levels of these parameters were observed between the treatment groups (Figure S2).

### Changes in RA treatment over time

**Fig. 5:**
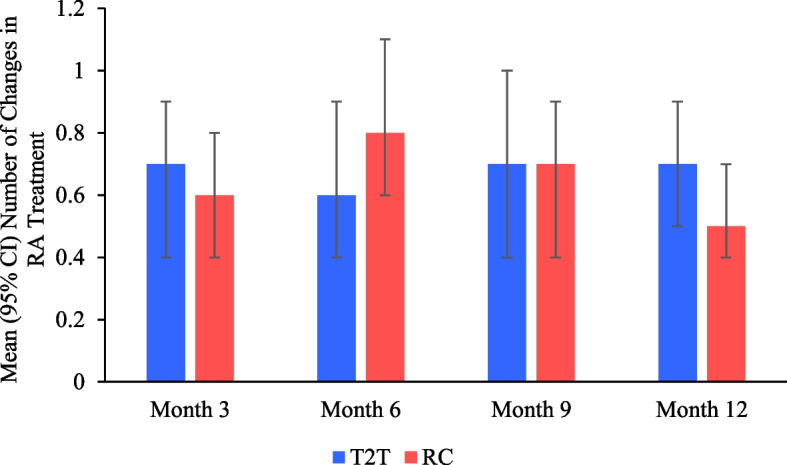
Mean number of changes in rheumatoid arthritis (RA) treatment through 1 year. At months 3, 6, 9, and 12, T2T sample size was 106, 93, 87, and 86, and RC sample size was 138, 136, 125, and 123, respectively. CI: confidence interval; RARC: routine care; T2T: treat-to-target

The proportion of patients remaining on abatacept treatment at 12 months was significantly lower in the T2T group compared with RC (51.5% vs. 66.9%, *p* = 0.027; data not shown). The mean number of changes in RA treatment throughout the study was comparable between the regimens with 0.7, 0.6, 0.7, and 0,7 changes in T2T, and 0.6, 0.8, 0.7, and 0.5 changes in RC at months 3, 6, 9, and 12, respectively (Fig. 5). Compared with RC, a lower proportion of patients in the T2T group had adjustments in MTX at month 3 (5.7% vs. 13.0%), 6 (9.6% vs. 17.6%), 9 (9.2% vs. 12.0%), and 12 (9.3% vs. 17.9%), and a higher proportion had changes in NSAIDs at month 3 (7.5% vs. 6.5%), 6 (9.6% vs. 5.1%), 9 (11.5% vs. 6.4%), and 12 (10.5% vs. 4.1%; data not shown). Changes in all other types of non-biologic RA medications, including corticosteroids, were similar between the regimens (data not shown).

### Safety

Rates of AEs and SAEs were generally low and comparable between treatment groups (Table S2). During the course of the study, in the overall population, AEs were reported for 189 (66.5%) patients and SAEs for 28 (9.9%) patients. The most frequent AEs were infections and infestations (34.5%), of which the most common (7.7%) was upper respiratory tract infection. Nine (3.2%) patients had a neoplasm, and 64 (22.5%) experienced general disorders and administration site conditions including 39 (13.7%) patients reporting the drug as ‘ineffective’. Details of AEs are provided in Table S3.

## Discussion

The ABC study compared the effectiveness of the T2T disease management strategy with RC among adult RA patients with moderate to severe disease activity who initiated treatment with SC abatacept. Over the course of 1 year, approximately 40% of patients managed according to either strategy achieved sustained LDA, as defined by CDAI ≤ 10, with a comparable time to achievement of this outcome in both treatment groups. Similar proportions of patients in the T2T and RC groups achieved LDA, remission, and improved physical function, as assessed by various clinical and PRO measures. No differences were observed in the likelihood of achieving these outcomes between the regimens, with the exception of patients managed per T2T who had 1.3 times higher odds of achieving CDAI LDA. Time to achieve SDAI remission was significantly shorter in the T2T group compared with RC and although no statistically significant differences were observed in the time to achieve other clinical and PRO measures, a trend toward a more rapid response in CDAI LDA and remission, DAS28-CRP remission, and HAQ-DI MCID, but not RAPID3 LDA and remission, was noted in the T2T group compared with RC. Previous studies have suggested that RAPID3 alone may not be sufficient to follow long-term disease activity in patients with RA in clinical practice and that it may capture aspects of RA other than inflammation [33, 34]. Thus, while in the present study RAPID3 may add valuable information on patient psychometrics, its utility in the assessment of disease activity may be limited.

The T2T strategy is the preferred RA management regimen as opposed to usual care [20, 21] with current treatment guidelines recommending implementation of T2T for all RA patients [6, 7]. However, the present study found that outcomes across validated measures of clinical RA disease activity and physical function were largely similar between patients managed per T2T and RC. One explanation for the lack of distinction between the regimens may be abatacept efficacy. Alternatively, treating physicians in the RC group may have been practicing according to the T2T strategy. The Canadian treatment guidelines advocating for T2T patient management were first published in 2012 [23], which may have had an impact on usual care. The similar number of treatment adjustments made over time in both groups in the present study further supports this possibility. However, although T2T awareness among physicians has grown, variable rates of implementation of and adherence to T2T guidelines in real-world daily clinical practice have been reported [35–37]. Patient-related factors such as comorbidities, communication barriers, side effects that preclude treatment escalation, and individual patient preferences may hinder the application of T2T recommendations [22]. Similarly, healthcare structures, clinical time constraints, lack of electronic reporting efficiency, and access to bDMARDs may result in deviations from T2T guidelines [22]. In the Canadian setting, however, support for and application of T2T recommendations was generally high among rheumatologists surveyed and action plans to encourage the adoption and application of the T2T recommendations are ongoing [38].

Despite a comparable number of changes in RA medications between treatment groups in the current analysis, physicians employing RC tended to adjust the use of MTX more frequently than those following T2T, who in turn were more likely to modify NSAIDs or discontinue abatacept presumably to switch to a different bDMARD. These treatment differences may have impacted patient outcomes, possibly accounting for the similarities between the regimens. Additionally, the T2T strategy is known to be more effective than RC in early RA [39, 40] while patients in the present study had established disease where, as previously demonstrated, achievement of therapeutic endpoints is not affected by implementation of a T2T strategy [41].

Overall, SC abatacept was found to be safe and well-tolerated, with no new safety concerns identified, consistent with the known abatacept safety profile. Over half of all patients experienced TEAEs, the majority of which were mild, with approximately 10% experiencing serious AEs.

The present study had some limitations. Enrollment of physicians who were already applying the T2T approach in their medical practice could lead to selection bias. Physicians were informed of the objectives of the study, though not the specific T2T plan to be followed, potentially influencing their therapeutic approach during the study. Further, since treatment adjustments were not necessarily made using disease activity as an outcome measure, each investigator may have used a different target definition, therefore it is not possible to assess the impact of T2T education on disease management. Information on the intensity of the treatment prior to study enrollment, including management approach was not examined. Given that data were collected as per usual care, there was considerable missing data for some variables, such as anti-CCP status. At the time of study design, the relationship between response to abatacept and seropositivity was not documented; therefore, this data was not mandated in the protocol. Furthermore, this test is not universally reimbursed in some provincial healthcare systems. It would, however, be assumed that many RF-positive patients were also CCP-positive and that due to randomization, the rates of positivity should be similar in the two treatment groups. Strengths of the present study include the real-world clinical setting, minimal exclusion criteria, and no intervention in patient management other than the provision of T2T training in the T2T group, rendering results with high external validity. Several validated clinical and PRO measures were utilized allowing for consistent assessment of disease outcomes.

## Conclusions

In this cohort of patients with moderate to severely active RA, SC abatacept was effective and well-tolerated, irrespective of the treatment approach used to manage the disease. The T2T approach may offer a greater likelihood of achieving CDAI LDA as well as faster achievement of SDAI remission.

### Supplementary Information

**Additional file 1.**

## Data Availability

Data can be made available upon request. Bristol Myers Squibb’s policy on data sharing may be found at https://www.bms.com/researchers-and-partners/independent-research/data-sharing-request-process.html.

## Abbreviations

ABC: Abatacept Best Care
AE: Adverse event
b: Biologic
CDAI: Clinical Disease Activity Index
CI: Confidence interval
CRP: C-reactive protein
DAS28-CRP: Disease Activity Score 28-CRP
DMARD: Disease-modifying anti-rheumatic drugs
HAQ-DI: Health Assessment Questionnaire Disability Index
HRQoL: Health-related quality of life
JAKi: Janus kinase inhibitor
K-M: Kaplan–Meier
LDA: Low disease activity
LSM: Least squares mean
MCID: Minimum clinically important difference
MDGA: Physician global assessment
mITT: Modified intent-to-treat
MMRM: Mixed models for repeated measures
MTX: Methotrexate
NSAID: Non-steroidal anti-inflammatory drugs
OR: Odds ratio
PRO: Patient reported outcome
PtGA: Patient global assessment
RA: Rheumatoid arthritis
RAPID3: Routine Assessment of Patient Index Data 3
RC: Routine care
SAE: Serious adverse event
SC: Subcutaneous
SD: Standard deviation
SDAI: Simple Disease Activity Index
SE: Standard error
SJC: Swollen joint counts
T2T: Treat-to-target
TEAE: Treatment-emergent adverse event
TJC: Tender joint counts
ts: Targeted synthetic
VAS: Visual analogue scale
