# Supplementary material for: Effectiveness of a treat-to-target strategy in patients with moderate to severely active rheumatoid arthritis treated with abatacept
Source: Arthritis Res Ther. 2023 Sep 28;25:183. doi: 10.1186/s13075-023-03151-2 (PMC10537125; doi:10.1186/s13075-023-03151-2)
Supplement: Supplementary file 1 — Additional file 1. [file 13075_2023_3151_MOESM1_ESM.docx]

# Supplementary Tables/Figures

Supplementary Table S1. Prior and concomitant rheumatoid arthritis medications

|  | **Treat-to-Target**  **(N=130)** | **Routine Care**  **(N=154)** | **Total**  **(N=284)** |
| --- | --- | --- | --- |
| **Prior bDMARD**, n (%) | | | |
| Any | 54 (41.5) | 57 (37.0) | 111 (39.1) |
| Adalimumab | 12 (9.2) | 14 (9.1) | 26 (9.2) |
| Infliximab | 4 (3.1) | 0 (0.0) | 4 (1.4) |
| Rituximab | 1 (0.8) | 0 (0.0) | 1 (0.4) |
| Golimumab | 10 (7.7) | 6 (3.9) | 16 (5.6) |
| Certolizumab | 9 (6.9) | 7 (4.5) | 16 (5.6) |
| Tocilizumab | 1 (0.8) | 1 (0.6) | 2 (0.7) |
| Etanercept | 18 (13.8) | 28 (18.2) | 46 (16.2) |
| Other medication | 1 (0.8) | 1 (0.6) | 2 (0.7) |
| Missing | 1 (0.8) | 0 (0.0) | 1 (0.4) |
| **Concomitant non-bDMARD**, n (%) | | | |
| Any | 118 (90.8) | 143 (92.9) | 261 (91.9) |
| Sulfasalazine | 13 (10.0) | 34 (22.1) | 47 (16.5) |
| Methotrexate | 91 (70.0) | 96 (62.3) | 187 (65.8) |
| Leflunomide | 23 (17.7) | 15 (9.7) | 38 (13.4) |
| Gold compound | 1 (0.8) | 1 (0.6) | 2 (0.7) |
| Corticosteroids | 24 (18.5) | 41 (26.6) | 65 (22.9) |
| NSAIDs | 39 (30.0) | 43 (27.9) | 82 (28.9) |
| Hydroxychloroquine | 47 (36.2) | 68 (44.2) | 115 (40.5) |
| bDMARD, biologic disease-modifying anti-rheumatic drugs; NSAID, Non-steroidal anti-inflammatory drugs | | | |

Supplementary Table S2. Summary of incidence of adverse events

|  | **Study Group** | |  |
| --- | --- | --- | --- |
|  | **Treat-to-Target**  **(N=130)** | **Routine Care**  **(N=154)** | **Total**  **(N=284)** |
| **System Organ Class**  Preferred Term | **n (%)** | **n (%)** | **n (%)** |
| **Any AE** | 80 (61.5) | 109 (70.8) | 189 (66.5) |
| **Any SAE** | 13 (10.0) | 15 (9.7) | 28 (9.9) |
| **Infections And Infestations** | 50 (38.5) | 48 (31.2) | 98 (34.5) |
| Upper Respiratory Tract Infection | 16 (12.3) | 6 (3.9) | 22 (7.7) |
| Bronchitis | 9 (6.9) | 13 (8.4) | 22 (7.7) |
| Sinusitis | 7 (5.4) | 8 (5.2) | 15 (5.3) |
| Pneumonia | 7 (5.4) | 2 (1.3) | 9 (3.2) |
| **Neoplasms Benign, Malignant and**  **Unspecified (Incl Cysts and Polyps)** | 3 (2.3) | 6 (3.9) | 9 (3.2) |
| **General Disorders and Administration Site**  **Conditions** | 25 (19.2) | 39 (25.3) | 64 (22.5) |
| Drug Ineffective | 16 (12.3) | 23 (14.9) | 39 (13.7) |
| Drug Effect Decreased | 1 (0.8) | 2 (1.3) | 3 (1.1) |
| Disease Progression | 0 (0.0) | 1 (0.6) | 1 (0.4) |

A patient may have reported more than one medical history. Percentages are based on the total number of enrolled patients in each category.

AE, adverse event; Incl, includes; SAE, serious AE

Supplementary Table S3. Incidence of adverse events in ≥5% of patients

|  | **Study Group** | |  |
| --- | --- | --- | --- |
|  | **Treat-to-Target**  **(N=130)** | **Routine Care**  **(N=154)** | **Total**  **(N=284)** |
| **System Organ Class**  Preferred Term | **n (%)** | **n (%)** | **n(%)** |
| **Overall** | 80 (61.5) | 109 (70.8) | 189 (66.5) |
| **Infections And Infestations** | 50 (38.5) | 48 (31.2) | 98 (34.5) |
| Upper Respiratory Tract Infection | 16 (12.3) | 6 (3.9) | 22 (7.7) |
| Bronchitis | 9 (6.9) | 13 (8.4) | 22 (7.7) |
| Sinusitis | 7 (5.4) | 8 (5.2) | 15 (5.3) |
| Nasopharyngitis | 6 (4.6) | 2 (1.3) | 8 (2.8) |
| Pneumonia | 7 (5.4) | 2 (1.3) | 9 (3.2) |
| **Musculoskeletal and Connective Tissue Disorders** | 28 (21.5) | 28 (18.2) | 56 (19.7) |
| Rheumatoid Arthritis | 8 (6.2) | 4 (2.6) | 12 (4.2) |
| **General Disorders and Administration Site Conditions** | 25 (19.2) | 39 (25.3) | 64 (22.5) |
| Drug Ineffective | 16 (12.3) | 23 (14.9) | 39 (13.7) |
| **Gastrointestinal Disorders** | 18 (13.8) | 22 (14.3) | 40 (14.1) |
| **Skin And Subcutaneous Tissue Disorders** | 25 (19.2) | 17 (11.0) | 42 (14.8) |
| Rash | 9 (6.9) | 3 (1.9) | 12 (4.2) |
| **Nervous System Disorders** | 20 (15.4) | 14 (9.1) | 34 (12.0) |
| Headache | 7 (5.4) | 4 (2.6) | 11 (3.9) |
| **Respiratory, Thoracic and Mediastinal Disorders** | 13 (10.0) | 13 (8.4) | 26 (9.2) |
| Asthma | 1 (0.8) | 0 (0.0) | 1 (0.4) |
| **Injury, Poisoning and Procedural Complications** | 8 (6.2) | 10 (6.5) | 18 (6.3) |
| **Investigations** | 4 (3.1) | 11 (7.1) | 15 (5.3) |

Supplementary Figure S2. Change from baseline in disease outcomes through 1 year. All between-group comparisons were p>0.05. At months 3, 6, 9, and 12, T2T sample size was 106, 94, 87, 86, and RC sample size was 138, 136, 125, and 123, respectively. CI, confidence interval; RC, routine care; SJC, swollen joint count; T2T, treat-to-target; TJC, tender joint count

**
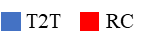
**
